# Risk of falling among hospitalized patients with high modified Morse scores could be further Stratified

**DOI:** 10.1186/s12913-017-2685-2

**Published:** 2017-11-13

**Authors:** Irina Gringauz, Yael Shemesh, Amir Dagan, Irina Israelov, Dana Feldman, Naama Pelz-Sinvani, Dan Justo, Gad Segal

**Affiliations:** 1 Internal Medicine T Department, Chaim Sheba Medical Center, 5265601 Tel-Hashomer, Israel; 2 Patient Safety Assurance Unit, Chaim Sheba Medical Center, Tel-Hashomer, Israel; 3 Geriatric Medicine D Department, Chaim Sheba Medical Center, Tel-Hashomer, Israel; 4 Sackler School of Medicine, Tel-Aviv University, Tel-Aviv, Israel

**Keywords:** Falls, Morse Fall Scale, Risk stratification, Hospitalization

## Abstract

**Background:**

Falls during hospitalization harbor both clinical and financial outcomes. The modified Morse fall scale [MMFS] is widely used for an in-hospital risk-of-fall assessment. Nevertheless, the majority of patients at risk of falling, i.e. with high MMFS, do not fall. The aim of this study was to ascertain our study hypothesis that certain patients' characteristics (e.g. serum electrolytes, usage of a walking device etc.) could further stratify the risk of falls among hospitalized patients with MMFS.

**Methods:**

This was a retrospective cohort analysis of adult patients hospitalized in Internal Medicine departments.

**Results:**

The final cohort included 428 patients aged 76.8±14.0 years. All patients had high (9 or more) MMFS upon admission, and their mean MMFS was 16.2±6.1. A group of 139 (32.5%) patients who fell during their hospitalization was compared with a control group of 289 (67.5%) patients who did not fall. The fallers had higher MMFS, a higher prevalence of mild dependence, and a greater use of a cane or no walking device. Regression analysis showed the following patients' characteristics to be independently associated with an increased risk of falling: mild dependence (OR=3.99, 95% CI 1.97-8.08; *p*<0.0001), treatment by anti-epileptics (OR=3.9, 95% CI 1.36-11.18; *p*=0.011), treatment by hypoglycemic agents (OR=2.64, 95% CI 1.08-6.45; *p*= 0.033), and hypothyroidism (OR=3.66, 05%CI 1.62-8.30; *p*=0.002). In contrast to their role in the MMFS, the use of a walker or a wheelchair was found to decrease the risk of falling (OR=0.3, 95% CI 0.13-0.69; *p*=0.005 and OR=0.25, 95% CI 0.11-0.59; *p*= 0.002).

**Conclusions:**

Further risk stratification of hospitalized patients, already known to have a high MMFS, which would take into account the characteristics pointed out in this study, should be attained.

## Background

Falling during hospitalization is a common phenomenon among hospitalized patients, becoming more frequent and hazardous among the elderly and the frail [1, 2]. Many efforts are being made worldwide to find the best means for fall-prevention and a better assessment of the risk of falling during a hospital stay [3]. Nevertheless, falls are still a major public-health concern [2]. Accordingly, there is an ongoing effort for the development of risk assessment tools in order to identify patients with an increased risk of falling as soon as they are hospitalized.

One of the commonly used risk assessment tools is the modified Morse fall scale [4, 5]. All of the patients at the Sheba Medical Center are assessed for a fall risk upon admission by the nurses who use a modified Morse fall scale (MMFS), as presented in Table 1. This version of the MMFS [4] consists of the patient's demographics, background diagnoses, therapeutic agents, functional status, cognition, and other characteristics potentially affecting the risk of falling. It is a detailed version of the MMFS that was adjusted to application in a major, tertiary medical center. For example, the "Use of major tranquilizers" in the MMFS was specifically translated to "undergoing general anesthesia during the previous 24 hours". Patients with high (9 or more) modified Morse fall scale scores are considered to have a higher risk of falling, and accordingly, preventive measures are used in these patients, including instructing them how to avoid obstacles in the hospital, which shoes to wear, and how to call a nurse when getting out of bed at night.

**Table 1:** A modified Morse scale for risk assessment used in the Chaim Sheba Medical Center

Category	Score for "Yes"	Score for "No"
Does the patient take more than 4 different medications?	1	0
Treatment with specific medications (cumulative score)		
Anti-histamine	1	0
Anti-hypertensive	1	0
Diuretics	1	0
Medications interfering with the state of alertness	1	0
Psychiatric medications	1	0
Laxatives	1	0
Anti-diabetic medications	1	0
Level of consciousness		
Mild confusion	1	0
Confused	1	0
Vaguely conscious	1	0
Unconscious	1	0
Sedated	1	0
Under the influence of medications	1	0
Cognitive state		
Difficulties in orientation, time	1	0
Difficulties in orientation, place	1	0
Difficulties in orientation, people	1	0
Memory problems	1	0
Vision		
Normal/Sedated	0	
Difficulties in vision	1	0
Eyeglasses all day long	1	0
Blindness	1	0
Hearing		
Normal/Sedated	0	
Difficulties in hearing	1	0
Hearing aid all day long		
Deafness	1	0
Mobility		
Fully independent/bedridden	0	
Needs little help	1	0
Needs significant help	1	0
Needs much help	1	0
Walking and stability		
Walks stably	0	
Weakness	1	0
Instability	1	0
Paralyzed	1	0
Bedridden	1	0
Wheelchair	1	0
Patient attached to limiting equipment (e.g., infusion set apparatus)	1	0
Use of mobility aids		
Does not use	0	
Cane	1	0
Walks with the help of the nursing staff	1	0
Walker	1	0
Wheelchair	1	0
Crutches	1	0
Amputation/Artificial limb	1	0
History of falling during the past 6 months	9	0
Difficulty in getting out of a bed or a chair	1	0
Patient feels weaker than before	1	0
Patient feels dizzy	1	0
Patient has decreased leg sensation	1	0
Did the patient undergo general anesthesia during the past 24 hours?	1	0
Urgency and a high frequency of the need to go to the toilet	1	0
Does the patient go to the toilet during the night-time?	1	0

Many patients, especially the elderly frail patients, are considered to be at a high risk of falling according to the modified Morse scale, but only a minority of these will actually fall during their hospital stay, as shown previously in sensitivity analysis (including specificity, positive predictive value (PPV), negative predictive value (NPV) and accuracy) [6]. Our goal, in the present study, was to ascertain our study hypothesis that certain patients' characteristics (some relating to demographics and co-morbidities like hypothyroidism and smoking status, some addressing laboratory parameters like sodium and potassium blood concentrations, some addressing chronic medication like anti-epileptics and some other clinical data like usage of a walking device; fully specified in Table 2) could further stratify the risk of falls among hospitalized patients with MMFS.

**Table 2:** Clinical characteristics of the whole cohort

	Total (*n*=428)	Fallers (*n*=139)	Non-fallers (*n*=289)	Relative risk (95% confidence interval)	*p* value
Demographics and chronic co-morbidities					
Age, years, mean±SD	76.8±14.0	75.0±13.2	77.7±14.3	/	0.062
Men, *n* (%)	210 (49.1)	77 (55.4)	133 (46.0)	1.4 (0.9-2.1)	0.079
Hypertension, *n* (%)	278 (65.0)	89 (64.0)	189 (65.4)	0.9 (0.6-1.4)	0.829
Ischemic heart disease, *n* (%)	149 (35.0)	54 (39.1)	95 (33.0)	1.3 (0.8-1.9)	0.233
Atrial fibrillation, *n* (%)	100 (23.4)	26 (18.7)	74 (25.6)	0.6 (0.4-1.1)	0.143
Diabetes mellitus, *n* (%)	163 (38.1)	59 (42.4)	104 (36.0)	1.3 (0.8-1.9)	0.204
Dementia, *n* (%)	66 (15.4)	17 (12.2)	49 (17.0)	0.6 (0.3-1.2)	0.253
Congestive heart failure, *n* (%)	103 (24.1)	36 (25.9)	67 (23.2)	1.1 (0.7-1.8)	0.548
Chronic renal failure, *n* (%)	142 (33.3)	55 (39.6)	87 (30.2)	1.5 (0.9-2.3)	0.062
Post-stroke, *n* (%)	91 (21.3)	28 (20.1)	63 (21.8)	0.9 (0.5-1.4)	0.801
Chronic lung disease, *n* (%)	45 (10.5)	19 (13.8)	26 (9.0)	1.6 (0.8-3.0)	0.177
Peripheral vascular disease, *n* (%)	28 (6.6)	8 (5.8)	20 (6.9)	0.8 (0.3-1.9)	0.835
Cancer, *n* (%)	93 (21.7)	29 (20.9)	64 (22.1)	0.9 (0.5-1.5)	0.803
Parkinson's disease, *n* (%)	21 (4.9)	4 (2.9)	17 (5.9)	0.4 (0.1-1.4)	0.236
Hypothyroidism, *n* (%)	42 (9.8)	19 (13.8)	23 (8.0)	1.8 (0.9-3.5)	0.081
Smoking, *n* (%)	54 (12.6)	26 (18.8)	28 (9.7)	2.1 (1.2-3.8)	0.012
Laboratory					
Hemoglobin, g/dl, mean±SD	11.4±2.0	11.3±1.9	11.5±2.0	/	0.313
Albumin, g/dl, mean±SD	3.3±0.5	3.2±0.5	3.3±0.5	/	0.062
Calcium, mg/dl, mean±SD	8.8±0.7	8.7±0.7	8.9±0.7	/	0.008
Potassium, mEq/L, mean±SD	4.3±0.7	4.1±0.6	4.4±0.8	/	<0.0001
Urea, mg/dl, mean±SD	66.3±50.1	69.3±49.8	64.8±50.3	/	0.383
Creatinine, mg/dl, mean±SD	1.4±0.8	1.5±1.0	1.3±0.7	/	0.201
Alanine aminotransferase, IU, mean±SD	35.1±113.7	35.2±71.4	35.0±129.3	/	0.985
Sodium, mEq/L, mean±SD	135.6±5.2	135.7±4.3	135.6±5.6	/	0.778
Drugs					
Anti-Parkinson’s, *n* (%)	28 (6.5)	8 (5.8)	20 (6.9)	0.8 (0.3-1.9)	0.835
Benzodiazepines, *n* (%)	157 (36.7)	60 (43.2)	97 (33.6)	1.5 (0.9-2.2)	0.055
Diuretics, *n* (%)	156 (36.4)	48 (34.5)	108 (37.4)	0.8 (0.5-1.3)	0.593
Anti-epileptics, *n* (%)	26 (6.1)	15 (10.8)	11 (3.8)	3.0 (1.3-6.8)	0.008
Opiates, *n* (%)	71 (16.6)	21 (15.1)	50 (17.3)	0.8 (0.4-1.4)	0.677
Statins, *n* (%)	152 (35.5)	46 (33.1)	106 (36.7)	0.8 (0.5-1.3)	0.518
Oral hypoglycemics, *n* (%)	43 (10.0)	19 (13.7)	24 (8.3)	1.7 (0.9-3.3)	0.089
Insulin, *n* (%)	92 (21.5)	30 (21.6)	62 (21.5)	1.0 (0.6-1.6)	0.999
Nitrates, *n* (%)	28 (6.6)	8 (5.8)	20 (6.9)	0.8 (0.3-1.9)	0.835
Neuroleptics, *n* (%)	62 (14.5)	22 (15.9)	40 (13.8)	1.1 (0.6-2.0)	0.560
Steroids, *n* (%)	89 (20.8)	35 (25.4)	54 (18.7)	1.4 (0.9-2.4)	0.127
¥Antidepressants, *n* (%)	80 (18.7)	24 (17.4)	56 (19.4)	0.8 (0.5-1.4)	0.692
Other data					
Morse falls score, mean±SD	16.2±6.1	17.3±5.6	15.7±6.3	/	0.016
Came from nursing home, *n* (%)	85 (19.9)	21 (15.1)	64 (22.1)	0.6 (0.3-1.0)	0.094
Falls in the previous 6 months, *n* (%)	289 (67.5)	96 (69.1)	193 (66.8)	1.1 (0.7-1.7)	0.661
Functionally Independent, *n* (%)	105 (24.5)	27 (19.4)	78 (27.0)	/	<0.0001
Mildly dependent, *n* (%)	232 (54.2)	96 (69.1)	136 (47.1)	/	
Fully dependent, *n* (%)	91 (21.3)	16 (11.5)	75 (26.0)	/	
Walking device - no, *n* (%)	230 (53.7)	85 (61.2)	145 (50.2)	/	0.008
cane, *n* (%)	59 (13.8)	24 (17.3)	35 (12.1)	/	
walker, *n* (%)	70 (16.4)	17 (12.2)	53 (18.3)	/	
wheelchair, *n* (%)	69 (16.1)	13 (9.4)	56 (19.4)	/	

*SD* standard deviation; ¥Including SSRI and SNRI

## Methods

This was a historical prospective study conducted at the Sheba Medical Center, a large tertiary medical center. An institutional ethics committee, approved the study prior to its data collection. Need for consent was waived by the ethics committee.

The study group included all patients who had been admitted to all seven Internal Medicine departments at the Sheba Medical Center during 2013 who also had high modified Morse Fall Scale scores [5] upon admission and also fell during their hospitalization. In the case of readmissions, only the first admission was included in the analysis. The control group included random patients who had been admitted to a single internal medicine department (Internal Medicine T) at the Sheba Medical Center during 2013 who also had high modified Morse Scale scores upon admission but did not fall during their hospital stay. Falls definition was consistent with the Tinetti’s definition: “an event which results in a person coming to rest unintentionally on the ground or other lower level, not as a result of a major intrinsic event (such as stroke) or an overwhelming hazard” [7].

The patients' electronic medical charts were reviewed for the following data: age (years); male gender (yes/no); various chronic co-morbidities (yes/no); living in a nursing home (yes/no); walking device used (either cane/walker/wheelchair); independence in basic activities of daily living (BADL) according to the Katz index [8]; a history of falls during the six months prior to their admission; laboratory findings upon admission whose anomalies might be associated with weakness, falls, and frailty (including hemoglobin blood levels [9], albumin serum levels [10], urea and creatinine serum levels [11], Alanine aminotransferase serum levels [12], and electrolyte serum levels); a list of medications used during hospitalization which might be associated with weakness and falls (anti-Parkinson medications, benzodiazepines, diuretics, anti-epileptics, opiates, statins, oral hypoglycemic agents, insulin, nitrates, neuroleptics, steroids, anti-depressants [13–16]; and the modified Morse fall scale scores upon admission [5]. Fall events in this cohort, like all other hospitalized patients in our medical center, were reported within 24 hours of event to the unit of patient safety and risks management. This was the data base from which we recorded events of falling.

Continuous variables were expressed as a mean ± standard deviation, median, and inter-quartile range (IQR). The student's t-test was used to compare between the mean values of continuous variables with parametric distributions, and the Mann–Whitney test was used to compare between the mean values of continuous variables with non-parametric distributions. The Fisher's exact test was used to compare between the incidence and the prevalence of categorical variables. A multivariate binary logistic regression analysis with an enter method was used to study which variables were independently associated with in-hospital falls. All variables in the univariate analysis (Table 2) were included in the regression model (Table 3). A receiver-operating characteristic (ROC) curve was used to study how the modified Morse Fall Scale scores (of 9 or more) predict in-hospital falls. Thereafter, a second ROC curve was used to study how all the variables in Table 3 (including the modified Morse Fall Scale scores) together predict in-hospital falls. The area under the second ROC curve was calculated using the probability for in-hospital falls and the actual binary outcome (in-hospital falls).

**Table 3:** Binary regression analysis showing which variables were independently associated with increased and decreased risk of in-hospital falls

	Beta	Standard error	Odds ratio	95% confidence interval	p value
Associated with increased risk of falling					
Mildly dependent	1.385	0.359	3.99	1.97-8.08	<0.0001
Anti-epileptics	1.362	0.537	3.90	1.36-11.18	0.011
Hypothyroidism	1.300	0.417	3.66	1.62-8.30	0.002
Oral hypoglycemic	0.972	0.455	2.64	1.08-6.45	0.033
Morse falls score	0.069	0.025	1.07	1.01-1.12	0.007
Associated with decreased risk of falling					
Atrial fibrillation	−0.705	0.336	0.49	0.25-0.95	0.036
Potassium	−0.910	0.216	0.40	0.26-0.61	<0.0001
Walking with a walker	−1.191	0.422	0.30	0.13-0.69	0.005
Using a wheelchair	−1.366	0.431	0.25	0.11-0.59	0.002

Two-tailed *p*<0.05 was considered statistically significant. The statistical analyses were carried out using the 24th version of the SPSS statistical software (SPSS Inc., Chicago, IL, USA).

## Results

The final cohort included 428 patients: 218 (50.9%) women and 210 (49.1%) men. The mean age was 76.8±14.0 years (median: 80 years; IQR: 70–86 years) and the mean Modified Morse falls scores was 16.2±6.1 (median: 16; IQR: 11–19). The three most prevalent chronic co-morbidities were: hypertension, ischemic heart disease, and diabetes mellitus. The baseline patients' characteristics are detailed in Table 2.

The study group included 139 (32.5%) patients (fallers) and the control group included 289 (67.5%) patients (non-fallers). Among fallers, most (n=95; 68.3%) of the patients fell in their room, and the rest fell in the bathroom or in the corridor. The mean admission-to-fall time was 6.1±16.9 days (median: 2 days; IQR: 1–6 days). All of the fallers were examined by a physician immediately following the event, and 16 (11.5%) patients also underwent imaging studies or were referred to further consultation.

Compared with non-fallers, fallers had higher modified Morse Scores, a higher prevalence of mild dependence, and a higher use of a cane or no walking device, a higher prevalence of smoking, lower calcium serum levels, lower potassium serum levels, and a higher rate of treatment with anti-epileptics.

**Fig. 1:**
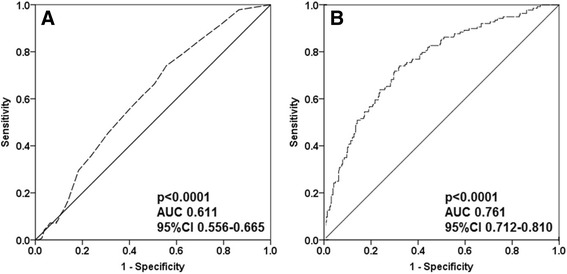
A receiver operating characteristic (ROC) curve analysis showing the Modified Morse Fall Scale scores prediction values before (**a**) and after (**b**) incorporation of all relevant variables

A regression analysis, as detailed in Table 3, showed that except for higher Morse Fall scores, mild dependence, the use of anti-epileptics, the use of oral hypoglycemic agents and hypothyroidism were independently associated with an increased risk of an in-hospital fall. On the other hand, in contrast to their role in the modified Morse scores, the use of a walker or a wheelchair was found to decrease the risk of falling (OR=0.3, 95% CI 0.13-0.69; *P* = 0.005 and OR=0.25, 95% CI 0.11-0.59; *P* = 0.002 respectively). Higher potassium serum levels and atrial fibrillation were also found to be independently associated with a decreased risk of an in-hospital fall. A receiver operating characteristic (ROC) curve analysis showed that Morse Fall Scale scores fairly predicts in-hospital falls even when they are 9 or higher (Fig. 1a). Nevertheless, According to the ROC curve presented, in patients with a modified MORSE scale score of 9 or more, the ideal cutoff to predict falls would be 14. The model that included all the variables presented in Table 3 even better predicted in-hospital falls (Fig. 1b).

## Discussion

Along with the substantial health sequel of falls, an enormous economic burden results from in-hospital falls and their direct consequences – particularly in the elderly population [17–20]. Accordingly, substantial efforts are being made world-wide to minimize the risk of falls during hospital stays [18]. A pillar of these efforts is the ability to predict upon the hospital admission the risk of falls for each admitted patient. The modified Morse scale is a widely-used tool for this reason and is thus, widely accepted. Nevertheless, we have noted that there is a huge population of patients which are classified, according to the modified Morse scale, as being at a high risk of falling, but only a minority of them actually fall during their hospital stay. Therefore, we have sought to further stratify this population of high modified Morse score patients (a version applicable to a tertiary, multidisciplinary medical center, as detailed previously in the introduction chapter) aiming at the isolation of the patients' characteristics which could improve our prediction of in-hospital falls.

Contrary to the modified Morse scale score equation, in the current analysis the use of a wheelchair or a walker has been associated with a decreased risk of falling during the hospital stay. This finding is surprising since in previous studies the use of a wheelchair or a walker has been associated with increased risk of falling [21], mainly in nursing-home residents [22]. It is possible, that during the hospital stay, their spouses and their caregivers look after them more intensively since they were using a wheelchair or a walker. This finding could be further investigated in the two following alternative ways: these variables could either have an opposite influence on the summation of a risk in the modified Morse scale score (a reduction of one point rather than adding one point to the total sum), or it could be omitted from the score. In either case, we assume that the modified Morse scale score could be more accurate than the way it is utilized today.

Atrial fibrillation, usually associated with impaired mobility in the elderly [23], is actually associated with reduced risk of falling in the current analysis. This unexpected finding is probably explained by the fact that most of these patients have been hooked-up to an over-head monitor during their hospital stay. The way that this fact should be incorporated into the risk score assessment should be further investigated and discussed.

Anti-epileptic drugs might be sedative and are associated with falls in the elderly [24] as are thyroid disorders [25]. According to our findings, the use of anti-epileptic drugs and hypothyroidism should be incorporated into the future developed scores for a risk assessment and accordingly, should be prospectively assessed.

The main limitation of the study is its small size - being a single-center study. These two drawbacks restrict the potential of generalizing our findings to larger patients' populations. In order to overcome this limitation, the study group has included all patients who have been admitted to all seven Internal Medicine departments at the Medical Center, and the control group has been twice larger. The current cohort size has allowed us to conduct a population-based multivariate analysis and still, larger cohorts should also be tested since it is possible that our final model overestimates the regression coefficients due to a low number of falls per independent variable. The retrospective nature of the study is another limitation due to a myriad of confounding factors that were not controlled in this type of study. Accordingly, prospective studies should be conducted in order to ascertain the findings of our research.

## Conclusions

The burden of comorbidities and the complex nature of both acute and chronic conditions for which elderly patients are admitted to hospitals are constantly growing. Therefore, it is only prudent to say that the problem of in-hospital falls will continue to grow. The parameters found in this study as potentially reducing or increasing the accuracy of the modified Morse score should be investigated in prospective studies.

## Abbreviations

IQR: Inter-quartile range
MMFS: Modified morse fall scale
ROC: Receiver operating characteristic
